# A Comparative Study on the Expression, Purification and Functional Characterization of Human Adiponectin in *Pichia pastoris* and *Escherichia coli*

**DOI:** 10.3390/ijms13033549

**Published:** 2012-03-15

**Authors:** Hussin A. Rothan, Ser Huy Teh, Kamariah Haron, Zulqarnain Mohamed

**Affiliations:** 1 Department of Molecular Medicine, Faculty of Medicine, University of Malaya, 50603, Kuala-Lumpur, Malaysia; E-Mail: molec.genetics@gmail.com; 2 Institute of Biological Science, Faculty of Science, University of Malaya, 50603, Kuala-Lumpur, Malaysia; E-Mail: serhuy@um.edu.my; 3 Center for Research in Biotechnology for Agriculture (CEBAR), University Malaya, 50603, Kuala-Lumpur, Malaysia; E-Mail: k_haron@um.edu.my

**Keywords:** adiponectin, *Pichia pastoris*, *E.coli*, recombinant protein, biological activity

## Abstract

Adiponectin is one of the most bioactive substances secreted by adipose tissue and is involved in the protection against metabolic syndrome, artherosclerosis and type II diabetes. Research into the use of adiponectin as a promising drug for metabolic syndromes requires production of this hormone in high quantities considering its molecular isoforms. The objective of this study is to produce recombinant human adiponectin by *Pichia pastoris* (P-ADP) as a cheap and convenient eukaryotic expression system for potential application in pharmaceutical therapy. For comparison, adiponectin was also expressed using the *Escherichia coli* (E-ADP) expression system. *Adiponectin* was constructed by overlap-extension PCR, and cloned in standard cloning vector and hosts. Recombinant expression vectors were cloned in the *P. pastoris* and *E. coli* host strains, respectively. SDS-PAGE and western blotting were used to detect and analyse expressed recombinant protein in both systems. Adiponectin was purified by affinity chromatography and quantified using the Bradford Assay. The results of this study indicated that P-ADP quantity (0.111 mg/mL) was higher than that of E-ADP (0.04 mg/mL) and both were produced in soluble form. However, P-ADP was able to form high molecular weights of adiponectin molecules, whilst E-ADP was not able to form isoforms higher than trimer. In addition, P-ADP was more active in lowering blood glucose compared with E-ADP. The two types of proteins were equally efficient and significantly decreased blood triglyceride and increased high density lipoprotein. We conclude that *P. pastoris* is able to produce high quantity of bioactive adiponectin for potential use in treatment of metabolic syndromes.

## 1. Introduction

Adipose tissue has been shown to play an important role in the regulation of body energy homeostasis and metabolism, lipid storage and as an endocrine organ. One of the most important factors secreted by adipose tissue is adiponectin (ADP) [1]. There is obvious correlation between plasma ADP levels and metabolic syndrome [2–4] and various studies have shown a noticeable decrease in ADP levels in patients with obesity, type II diabetes and with the accumulation of visceral adipose tissue [5–7]. In recent years, ADP has attracted much consideration as a novel therapeutic tool for diabetes and metabolic syndromes.

It is generally accepted that the choice of heterologous protein expression system has profound influence on characteristics of the recombinant protein [8]. Compared to prokaryotic expression systems like *Escherichia coli*, the advantages of eukaryotic expression system such as the methylotrophic yeast *Pichia pastoris* are many. These advantages include an efficient recombinant protein secretion pathway for ease of purification, the availability of eukaryotic post-translational modifications, fast growth on economic salt-based media and little risk of contamination with endo-toxins or oncogenic or viral DNAs [9]. Exclusively, *P. pastoris* has a very low maintenance energy demand, which makes it well suited for high cell density fermentation. The availability of strong and tightly regulated promoters makes this yeast a very attractive host for recombinant protein production [10].

Different groups have reported expression of different versions of human ADP in various types of host. The globular domain of ADP have been successfully expressed in *E. coli* and *P. Pastoris* [11,12] whilst the full-length protein have been expressed in *E. coli* and in baculovirus based expression systems [13]. Each reported varying degree of success in terms of yield and functionality of the recombinant protein. However, the expression of recombinant adiponectin in *P. pastoris* and evaluation of its activity have not been fully investigated. Therefore, in this study we examined the activity of recombinant adiponectin which was produced by *P. pastoris* (P-ADP) compared with that produced by *E. coli* (E-ADP). The results of this study showed that P-ADP underwent post translation modification and has better bioactivity than E-ADP. In addition, *P. pastoris* expression system was efficient in producing high quantity and quality of biologically active recombinant ADP compared with *E. coli* expression system.

## 2. Results

### 2.1. Construction and Cloning of Human *ADP* in *P. pastoris* and *E. coli*

**Figure 1:**
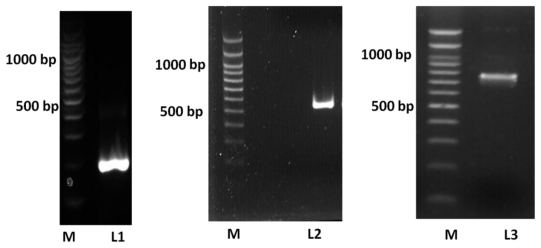
*In vitro* construction of *ADP* fragment using overlap-extension PCR. ADP fragment was amplified by PCR through 32 cycles include denaturing step (95 °C for 45 s), annealing step (60 °C for 45 s) and elongation step (72 °C for 1 min). The two fragments were then purified and joined through 10 cycles of overlap-extension PCR include denaturing step at 95 °C for 45 s, annealing step at 60 °C for 45 s and elongating step at 72 °C for 1 min. **L1**: PCR product of exon 2 (204 bp). **L2**: PCR product of exon 3 (531 bp). **L3**: the full length of *ADP* fragment (734 bp). **M**: 100 bp DNA marker.

The results of the overlap-extension PCR showed that the full-length *ADP* (735 bp) was successfully obtained using this procedure. The full length *ADP* encoded variable, collagenous and globular domains of ADP (Figure 1). Cloning of *ADP* downstream of the signal peptide and *MBP* sequences was successful in producing MBP-E-ADP periplasm fusion protein. Similarly, cloning of *ADP* in pPICZαA vector after the α-factor signal peptide was successful in producing 6× His tagged P-ADP that would eventually be secreted extracellularly.

### 2.1. Expression of ADP in *E. coli* and Protein Purification

**Figure 2:**
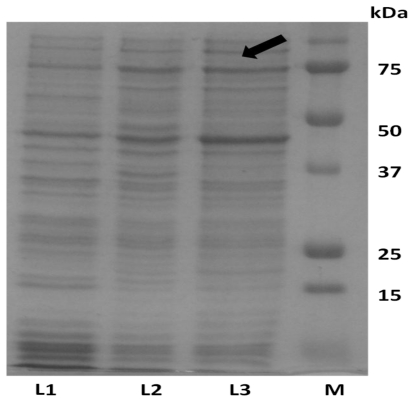
SDS-PAGE analysis of adiponectin expression before and after induction with IPTG. The expected size of fusion protein (MBP-adionectin) was approximately 75 kDa and this can be seen in reference to the protein marker (**M**). The band of fusion protein was faint before induction (**L1**). However, after induction with IPTG, the fusion protein was more detectable (arrow) after 2 h (**L2**) and 3 h (**L3**).

**Figure 3:**
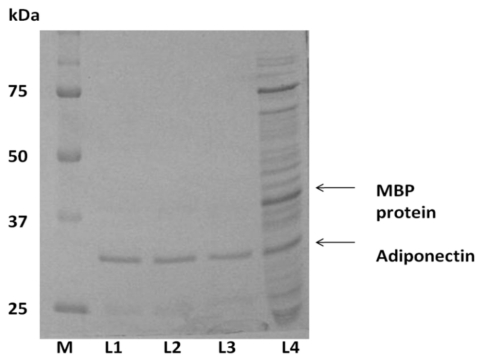
SDS-PAGE for optimization of Factor Xa digestion. This figure showed that there was no clear difference between protein digestion with Factor Xa for 2 h (**L1**), 4 h (**L2**) and 8 h (**L3**). Digestion of crude protein with Factor Xa showed that the expected size for maltose binding protein (MBP) is approximately 45 kDa whereas the expected size of adiponectin protein is approximately 30 kDa (**L4**).

*ADP* was inserted downstream of the *mal E* of *E. coli* that encodes the maltose binding protein (MBP) resulting in the expression of an MBP fusion protein. As such, the amylose resin column system was used for purification of this fusion protein. SDS-PAGE results indicated that the expected size of the fusion protein was approximately 75 kDa (Figure 2). As mentioned previously, MBP was linked to adiponectin protein by four amino acids (ile-glu-gly-arg). This link can be recognized by Factor Xa for cleavage and separation of adiponectin from MBP. The expected size of adiponectin after digestion with Factor Xa was approximately 30 kDa (Figure 3).

It is shown that there was no clear difference between protein samples after digestion with Factor Xa for different time points. In the positive control that contains crude protein digested with Factor Xa, the estimated molecular weight of MBP was higher than that of the adiponectin protein and this protein, like other *E. coli* native proteins, was totally removed after second purification by amylose resin column (Figure 3). The second purification was carried out to eliminate MBP residues. This purification was performed by passing the fusion protein cleavage product through the hydroxyapatite column to remove maltose residues. The eluted protein was then loaded into the amylose resin column and the flow through factions were collected. Protein in the flow through was free of MBP protein and consists of adiponectin protein with a total amount of 0.04 mg/mL (Figure 3).

### 2.3. Expression of ADP in *Pichia pastoris* and Protein Yield Optimization

**Figure 4:**
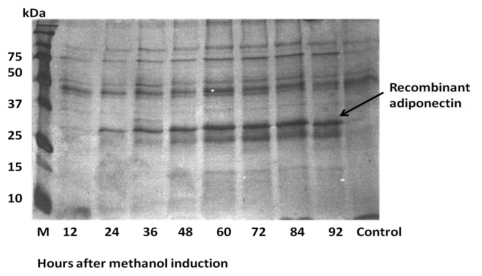
SDS-PAGE of time-course expression shows the exponential increase in band (arrow) densities starting from 12 h to 60 h of P-ADP expression after methanol induction.

**Figure 5:**
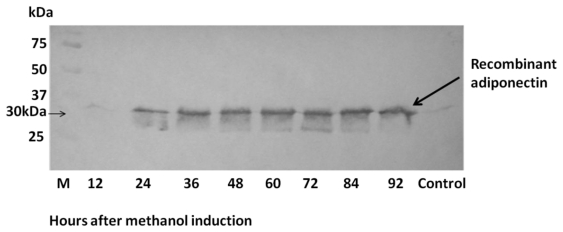
P-ADP molecules were detected by western blot analysis for time course expression using adiponectin monoclonal antibody.

A time course study was performed to determine optimal methanol induction times, maximum yeast growth rate and the best time for protein harvesting (Table 1). SDS-PAGE results showed that the expression of adiponectin protein was detected from 12 h after the beginning of methanol induction until the 96th hour of induction (Figure 4). Western blot analysis corroborated PAGE results with the estimated recombinant protein size of approximately 30 kDa (Figure 5). The highest concentration of expressed P-ADP protein was 0.11 mg/mL obtained at 60 h after the start of methanol induction (Table 1). P-ADP with 6× His tag was subsequently purified by one step affinity chromatography nickel column.

### 2.4. Analysis of Recombinant Adiponectin Protein Produced by *E.coli* and *P.pastoris*

**Figure 6:**
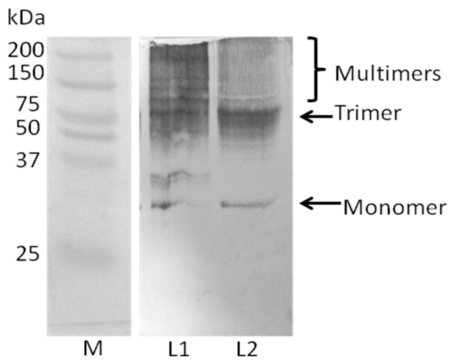
SDS-PAGE without denaturing condition showed less oligomers for adiponectin protein produced by *E. coli* compared with that produced by *Pichia pastoris.* M: Protein marker L1: Recombinant adiponectin produced by *Pichia pastoris.* L2: Recombinant adiponectin produced by *E. coli.*

SDS-PAGE under non-reducing condition was performed to detect the oligomerization process in adiponectin protein using anti-adiponectin anti-body. The expected size of the adiponectin monomer was approximately 30 kDa for each of the two types of protein. However, the recombinant adiponectin protein produced by *E. coli* showed less oligomers compared with adiponectin protein types that can be produced by *P. pastoris* (Figure 6).

### 2.4. Comparison of Biological Activity Between P-ADP and E-ADP

**Figure 7:**
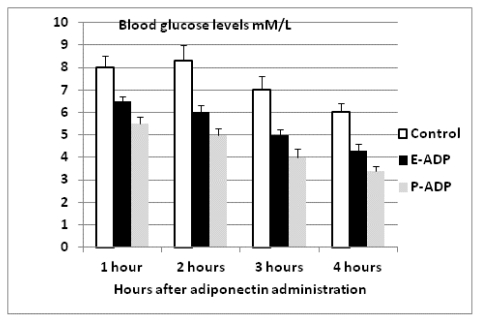
P-ADP and E-ADP significantly lowered blood glucose (*t*-test, *p* < 0.01) and there was significant difference (*t*-test, *p* < 0.05) between P-ADP and E-ADP in lowering blood glucose. Data were calculated by mean ± SEM.

**Figure 8:**
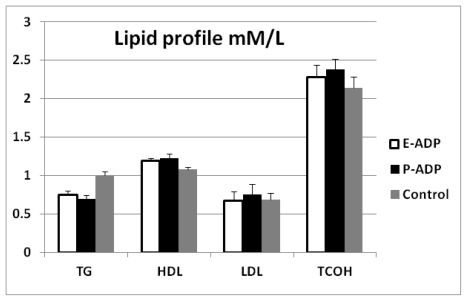
Effect of P-ADP and E-ADP on lipid profile after four hours from the first injection. The two types of protein significantly (*t*-test, *p* < 0.01) lowered the triglyceride levels, whereas these proteins caused significant increase (*t*-test, *p* < 0.05) in HDL levels. There were no significant effects on total cholestrol levels or LDL levels. Also, there were no significant differences between P-ADP and E-ADP. Data were calculated by mean ± SEM.

Comparison of biological activity between P-ADP and E-ADP showed that both types of recombinant protein significantly lowered blood glucose throughout the experiment period (Figure 7). Additionally, it was also observed that both types of proteins have significant effects on blood lipids by decreasing triglyceride levels and increasing HDL levels at the end of the experiment period. However, both these proteins showed no significant effect on total cholesterol and LDL levels (Figure 8). It is also important to note that P-ADP was significantly more active in lowering blood glucose comparing with E-ADP on the experiments conducted above.

## 3. Discussion

It has been shown previously that adiponectin functions as regulators of insulin sensitivity, glucose homeostasis and lipid metabolism [14–16]. The failure of adiponectin monomers to assemble into trimers would result in impaired secretion from the cell, and subsequently results in the diabetic phenotype with hypoadiponectinemia, as shown to be true for some adiponectin mutants [17]. Therefore, there is a need to be able to produce appropriately functional adiponectin in high quantities for pharmaceutical purposes, using cheap and convenient expression systems.

It is a general fact that quantity and quality of the recombinant protein as well as the cost of production are important considerations in producing pharmaceutical therapies. The recombinant proteins which are produced by different host cells vary in protein characteristic such as effective translation due to codon specificity, post-translational modifications, as well as the size of the resulting protein which is dependent on the degree of glycosylation [8]. For instance, certain recombinant proteins expressed in *E. coli* required extra processing steps for denaturation and refolding, in order to obtain biologically active forms [18]. Furthermore, recombinant proteins which are produced inside or outside host cells differ in the extent of accumulation in inclusion bodies [18]. In previous studies, recombinant globular domain of human adiponectin produce by *P. pastoris* was biologically active [12]. However, it was also reported that the full length protein showed higher activity than the shorter form (globular domain) [19]. Globular domain adiponectin is also limited in its ability to induce cellular response, as it had been shown to only affect one of two types of adiponectin receptors [20]. In view of that, we chose *P. pastoris* as a cheap alternative expression system to extracellularly produce full length adiponectin, which are more effective biomolecules, without the need for refolding treatments or additional steps of purification. Adiponectin protein produced by *E. coli* showed good solubility, but the level of post translation modification was lower than that of *P. pastoris*. The MBP tag was important in facilitating the production of soluble protein by *E. coli*. However, there would then be the requirement of additional purification steps to remove MBP. This is definitely disadvantageous compared to one step purification in the case of expression in *P. pastoris.*

In this study, two important factors facilitated high quantity production of ADP by *P. pastoris*. Firstly, the expression of ADP was driven by the strong and tightly regulated AOX1 promoter. Secondly, a time-course study of adiponectin expression enabled us to determine the specific time of high expression of recombinant protein and low proteolytic activity. The culture at sixty hours after methanol induction yielded 0.111 mg/mL, and this is a reasonably good quantity compared with that produced by *E. coli* (0.1 mg/mL [11], 0.04–0.08 mg/mL [13]), 0.01 mg/mL by baculovirus [13] and 0.05 mg/mL globular adiponectin expressed by *P. pastoris* [12]. The SDS-PAGE and western blotting analysis established that the approximated size of human adiponectin protein obtained in this study was similar to those previously reported [11,17].

Consistent with previous findings [19,21] ADP was effective in lowering blood glucose and triglycerides. ADP as full length proteins has the ability to effectively influence both AdipoR1 and AdipoR2 receptors, which stimulate glucose uptake and fatty acids oxidation [22,23]. Likewise, ADP has also been shown to be beneficial on blood lipid profile through decreasing triglycerides and increasing HDL. As such, our findings and results from several other studies corroborate the suggestion that ADP potentially has anti-atherogenic properties [24–26].

## 4. Experimental Section

### 4.1. Culture Media

*Escherichia coli* strain JM109 and TB1 were cultured in standard LB broth and selective LB-agar. The selective LB-agar medium contains 2% agar, 0.5 mM isopropylthiogalactoside (IPTG), 80 μg/mL of 5-bromo-4-chloro-3-indolyl-β-d-galactopyranoside (X-Gal) and 100 μg/mL ampicillin. *E. coli* strain TOP10 was cultured in low salt LB medium (LSLB) and LSLB-Agar with zeocin™ (salt concentration < 90 mM and pH 7.5 for zeocin to be active). LSLB medium contains 1% peptone, 0.05% NaCl, 0.5% yeast extract and 1.5% agar with 25 μg/mL Zeocin™ for solid medium.

*Escherichia coli* expression strain TB1 were cultured in LB rich medium containing 100 μg/mL ampicillin, 1% peptone, 0.5% yeast extract, 0.5% NaCl and 0.2% dextrose. Whereas, *P. pastoris* were cultured in YPD broth, containing 2% peptone, 1% yeast extract, 2% dextrose and 100 mg/L Zeocin™ YPDS-agar was made by including 2% agar and 18% sorbitol. For gene expression purposes, the BMMY and BMGY media was used. These buffered complex media contained 2% peptone, 1% yeast extract, 4 × 10^−5^ % biotin, 1.34% yeast nitrogen base, 0.1 M potassium phosphate buffer, pH 6.0 and 1% glycerol for BMGY growth medium, or 1% methanol for BMMY induction medium.

### 4.2. Gene Construction and Cloning

Adiponectin gene reference sequence was obtained from GenBank under the reference number NC_000003.11. Exon 2 and exon 3 of the ADP fragment was amplified individually by polymerase chain reaction (PCR). The PCR conditions through 32 cycles were 95 °C for 45 s as denaturing step, 60 °C for 45 s as an annealing step and 72 °C for 1 min as an elongation step. The two fragments were then joined by overlap-extension PCR. The reaction conditions of an overlap-extension PCR through 10 cycles were denaturing step at 95 °C for 45 s, annealing step at 60 °C for 45 s and elongating step at 72 °C for 1 min. The pMAL™-p4 vector (New England Biolabs, UK) was used to produce ADP in *E. coli* periplasm. This vector was digested with *Xmn* I and *Hind* III restriction enzymes (Promega, USA). At the same time, ADP fragment which prepared to express by *E. coli* was digested with *Hind* III restriction enzyme. The ligation mixture was prepared by adding digested vector and digested ADP fragment with DNA ligase and its suitable ligation buffer (New England Biolabs, UK).

*Adiponectin* fragment was cloned in pGEM^®^-T cloning vector (Promega, USA). After sequence verification, the recombinant plasmid was double digested with *Eco*RI and *Not*I restriction enzymes to generate fragments with cohesive termini. Digested fragments were purified using the QIAquick Gel Extraction kit (Qiagen, USA), as described in the manufacturer’s protocol. To clone in *P. pastoris*, ADP fragment were then ligated to the pPICZαA plasmid which had been digested with *Eco*RI and *Not*I, followed by phenol-chloroform extraction and ethanol precipitation. Following sequence verification, the pPICZαA-ADP recombinant plasmid was linearized with *Sac*I and transformed into competent X-33 *P. pastoris* cells using the EasyComp™ (Invitrogen, Netherlands) procedure.

### 4.3. Expression in *E. coli*

Single colony of cells containing fusion plasmid was used to inoculate 10 mL of LB broth and grown overnight at 37 °C. An overnight culture was used to inoculate one litre of an expression medium. The subculture was grown at 37 °C with good aeration (250 rpm shaking) until OD_600_ was approximately 0.5. An aliquot sample of 1 mL was taken as non-induced cells and centrifuged for 2 min. Then, cell pellet was resuspended in 50 μL of 1X SDS-PAGE sample buffer and frozen at −20 °C. For induction, IPTG was added to the remaining culture to a final concentration of 0.3 mM and the culture was incubated at 37 °C and 250 rpm shaking for 4 h. At each hour after induction a sample of 1 mL was taken and prepared for SDS-PAGE analysis.

### 4.4. Expression and Optimization of Adiponectin Production by *P. pastoris*

The recombinant X-33 single colony was inoculated in BMGY medium and grown at 30 °C until OD_600_ was 2–6. The cell pellet was resuspended in BMMY media (or BGMY medium for control culture) at OD_600_ = 1, and grown at 30 °C with shaking at 220 rpm. Methanol induction was carried out at 12 h intervals to a final concentration 0.5%, and harvesting was carried out at 12, 24, 36, 48, 60, 72, 84 and 96 h after induction. Glycerol was added to the BMGY medium as a substitute for methanol. Supernatants were collected from harvested cultures and secreted proteins were analysed by SDS-PAGE and western blot using anti-ADP antibody. In another experiment, five tubes containing 10 mL YPD medium were inoculated with single recombinant X-33 colony and incubated for 16–18 h until OD_600_ reached 2 to 6. For growth phase culture, the harvested cells were transferred to five 250 mL flasks containing 50 mL BMGY media and cultured as previously described until OD_600_ reached 2 to 6. Next, the harvested cells were resuspended in five 1 L flasks containing 200 mL BMMY media, and cultured until OD_600_ = 1. For induction phase, 100% methanol was added to a final concentration of 0.5% every 12 h for 60 h at 30 °C in a shaking incubator (250–300 rpm). To precipitate the secreted P-ADP, three volumes of acetone were added to the collected supernatants followed by incubation at −20 °C overnight. The dissolved protein was resuspended with PBS buffer, subsequently purified by affinity chromatography and quantified using the Bradford method.

### 4.5. Protein Purification

#### 4.5.1. E-ADP Purification by Amylose Resin Column

The amylose resin was poured into a disposable polypropylene column of 25 mL volume (BioRad Econo-Pac™, U.S.A.). The bed volume was 5 mL and the column volume was 20 mL. The column was washed with 8 column volume of column buffer (20 mM Tris-HCl, 200 mM NaCl and 1 mM EDTA). Then, the crude extract was loaded into the column at a flow rate about 1 mL/min. The column was turned off for 15 min to enable optimal binding between the fusion protein and the amylose resin. Later, the column was washed with 12 column volume of column buffer. In order to elute the bound protein, one column volume of elution buffer (column buffer with 10 mM maltose) was added and the fractions (3 mL) were collected and kept at −80 °C.

#### 4.5.2. Cleavage, Denaturing and Re-Purification of E-ADP

First, a pilot experiment was carried out using small portion of protein sample to optimize the suitable time for Factor Xa ( Biolabs, UK) cleavage. Factor Xa final concentration of 1% was added to the protein sample, the positive control was a protein sample before purification (crude protein) incubated with same concentration of Factor Xa. All samples were incubated for 2, 4 and 8 h. From each reaction 5 μL were added to 5 μL of 2× SDS-PAGE sample buffers and saved at 4 °C. SDS-PAGE was applied to determine the suitable time for cleavage. Then, the pilot experiment was scaled up for the portion of the fusion protein to be cleaved. Guanidine hydrochloride was added to the sample to a final concentration of 6 M. Later, the sample was dialysed against 100 sample volumes column buffer (20 mM Tris-HCl, 200 mM NaCl and 1 mM EDTA) three times for 2 h each. In order to remove the rest of maltose binding protein, the fusion protein cleavage mixture was loaded onto the hydroxyapatite column (BioRad Econo-Pac™, U.S.A.). The column was washed with 80 mL of 20 mM sodium phosphate, 200 mM NaCl (pH 7.2). The protein mixture was eluted with 0.5 M Na phosphate (pH 7.2). The collected fractions were loaded onto amylose column and the flow through was collected that should be free of maltose binding protein (MBP).

#### 4.5.3. P-ADP Purification by Nickel Column

Protein samples were purified by His GraviTrap™ Flow (Amersham Biosciences, USA) column containing precharged Ni Sepharose™ 6 Fast. The column was normalized with 8 mL of phosphate buffer (20 mM sodium phosphate buffer and 500 mM NaCl, pH 7.4). The sample (4 mL) was loaded into the column and the column was washed with 10 mL of binding buffer (phosphate buffer containing 20 mM imidazole, pH 7.4). The recombinant protein was eluted with 4 mL of elution buffer (phosphate buffer containing 200 mM imidazole, pH 7.4).

### 4.6. SDS-PAGE and Western Immunoblotting

Purified P-ADP and E-ADP proteins were separated by SDS-PAGE in denaturing and non-denaturing (with and without heat and reducing agents) conditions. The proteins were then transferred onto polyvinylidene fluoride membrane as described elsewhere [17]. In brief, protein samples were loaded into 12% gels SDS-PAGE, and then transferred to a nitrocellulose membrane (1 h, 100 V). Following transfer, the membrane was blocked in Tris Buffered Saline with Tween-20 containing 50 g/L skimmed milk for 2 h, and then incubated with adiponectin monoclonal antibody (abcam, UK) for 2 h at room temperature. The strips were washed three times with Tris Buffered Saline (15 min each time) and then incubated with mouse anti-IGg antibody conjugated with alkaline phosphatase (Sigma, USA) for 2 h, washed again with Tris Buffered Saline as described previously, and finally developed with Western Blue^®^ stabilized substrate (Promega, USA).

### 4.7. Effect of Recombinant Adiponectin on Blood Glucose and Lipids

Female ICR mice were used to compare the biological activity of ADP expressed in *P. pastoris* and *E. coli*. Animals were obtained from the Animal House, Faculty of Medicine, University of Malaya in Kuala Lumpur (Ethics No. PM 07/05/2010 MAA (a) (R). Following overnight fasting, animals (3 groups, *n* = 6 each) were gavaged with high fat-sucrose diet. Immediately after feeding, the first group was injected with 0.9 mg/kg of bodyweight P-ADP. The second group was injected with 0.9 mg/kg of bodyweight E-ADP and the final group was injected with 0.3 mL saline for experiment control. After one hour of the first injection (or one hour after feeding) a second dose of treatment was given to all mice (the total amount of P-ADP and E-ADP being administered for each mouse = 1.8 mg/kg bodyweight). Blood glucose was measured with a glucometer at one hour intervals for four hours. In addition, blood concentration of triglyceride (TG), total cholesterol (CHOL) (Siemens, U.S.A.), low density lipoprotein (LDL) and high density lipoprotein (HDL) (Dade Behring, U.S.A.) were also measured at the end of the fourth hour of the experiment using available commercial kits.

### 4.8. Statistical Analysis

Values are expressed as means ± S.E.M. The mean value comparisons between two groups were performed using the *Student t test*. Significant differences were considered at *p* < 0.05.

## 5. Conclusions

It is interesting to note that the *P. pastoris* expression system was able to produce higher quantity with simple purification and higher bioactivity of ADP compared to the expression by *E. coli*. All these factors together make *P. pastoris* the favourable expression system to produce recombinant human adiponectin for treatment of type II diabetes and cardiovascular diseases.

## Figures and Tables

**Table 1:** The differences in the optical density reading, total cells mass and protein concentration after methanol induction.

Hours after methanol Induction	Culture density (OD_600_)	Total cells mass (g)	Protein concentration (μg/mL)
12	27.75	1.02	5
24	36.57	1.49	22
36	35.5	1.51	25
48	52.8	1.60	93
60	49.25	1.60	111
72	51.33	1.80	78
86	49.0	1.62	48
96	43.15	1.78	77

## References

[1] Scherer P., Williams S., Fogliano M., Baldini G., Lodish H.A. (1995). novel serum protein similar to C1q, produced exclusively in adipocytes. J. Biol. Chem.

[2] Vasseur F.D., Meyre D., Froguel P. (2006). Adiponectin, type 2 diabetes and the metabolic syndrome: lessons from human genetic studies. Expert Rev. Mol. Med.

[3] Jang Y., Chae J.S., Koh S.J., Hyun Y.J., Kim J.Y., Jeong Y.J., Park S., Ahn C.M., Lee J.H. (2008). The influence of the adiponectin gene on adiponectin concentrations and parameters of metabolic syndrome in non-diabetic Korean women. Clin Chim Acta.

[4] Heid I., Wagner S., Gohlke H., Iglseder B., Mueller J., Cip P., Ladurner G., Reiter R., Stadlmayr A., Mackevics V. (2006). Genetic Architecture of the *APM1* Gene and Its Influence on Adiponectin Plasma Levels and Parameters of the Metabolic Syndrome in 1727 Healthy Caucasians. Diabetes.

[5] Hotta K., Funahashi T., Arita Y., Takahashi M., Matsuda M., Okamoto Y., Iwahashi H., Kuriyama H., Ouchi N., Maeda K. (2000). Plasma concentrations of a novel, adipose-specific protein, adiponectin, in type 2 diabetic patients. Arterioscler. Thromb. Vasc. Biol.

[6] Matsuzawa Y., Funahashi T., Kihara S., Shimomura I. (2004). Adiponectin and Metabolic Syndrome *Arterioscler*. Thromb. Vasc. Biol.

[7] Diez J., Iglesias P. (2003). The role of the novel adipocyte-derived hormone adiponectin in human disease. Eur. J. Endocrinol.

[8] Marini G., Forno G., Kratje R., Etcheverrigaray M (2007). Recombinant human granulocyte-macrophage colony-stimulating factor: effect of glycosylation on pharmacokinetic parameters. Electron. J. Biotechnol.

[9] Romanos M.A., Scorer C.A., Clare J.J. (1992). Foreign gene expression in yeast: a review. Yeast.

[10] Jahic M., Rotticci-Mulder J., Martinelle M., Hult K., Enfors S. (2002). Modeling of growth and energy metabolism of *Pichia pastoris* producing a fusion protein. Bioprocess. Biosyst. Eng.

[11] Xiao-Bo H., Yu-Jian Z., Hui-Tang Z., Sheng-Li Y., Yi G. (2003). Cloning and expression of adiponectin and its globular domain and measurement of the biological activity *in vivo*. Acta Biochim. Biophys. Sin.

[12] Liu D.G., Liu H.L., Song T.J., Huang H.Y., Li X., Tang Q.Q. (2007). Functional expression of the globular domain of human adiponectin in *Pichia pastoris*. Biochem. Biophys. Res. Commun.

[13] Avides M., Domingues L., Vicente A., Teixeira J. (2008). Differentiation of human pre-adipocytes by recombinant adiponectin. Prot. Expr. Purif.

[14] Takahashi M., Arita Y., Yamagata K., Matsukawa Y., Okutomi K., Horie M., Shimomura I., Hotta K., Kuriyama H., Kihara S. (2000). Genomic structure and mutations in adipose-specific gene adiponectin. Int. J. Obes. Relat. Metab. Disord.

[15] Kondo H., Shimomura I., Matsukawa Y., Kumada M., Takahashi M., Matsuda M., Ouchi N., Kihara S., Kawamoto T., Sumitsuji S. (2002). Association of adiponectin mutation with type 2 diabetes: a candidate gene for the insulin resistance syndrome. Diabetes.

[16] Xita N., Georgiou I., Chatzikyriakidou A., Vounatsou M., Papassotiriou G., Papassotiriou I., Tsatsoulis A. (2005). Effect of adiponectin gene polymorphisms on circulating adiponectin and insulin resistance indexes in women with polycystic ovary syndrome. Clin. Chem.

[17] Waki H., Yamauchi T., Kamon J., Ito Y., Uchida S., Kita S., Hara K., Hada Y., Vasseur F., Froguel P. (2003). Impaired multimerization of human adiponectin mutants associated with diabetes: Molecular structure and multimer formation of adiponectin. J. Biol. Chem.

[18] Lilie H., Schwarz E., Rudolph R. (1998). Advances in refolding of proteins produced in *E. coli*. Curr. Opin. Biotechnol.

[19] Pajvani U., Du X., Combs T., Berg A., Rajala M., Schulthess T., Engel J., Brownlee M., Scherer P. (2003). Structure-Function Studies of the Adipocyte-secreted Hormone Acrp30/Adiponectin. J. Biol. Chem.

[20] Kadowaki T., Yamauchi T., Kubota N., Hara K., Ueki K., Tobe K. (2006). Adiponectin and adiponectin receptors in insulin resistance, diabetes, and the metabolic syndrome. J. Clin. Invest.

[21] Kadowaki T., Yamauchi T. (2005). Adiponectin and adiponectin receptors. Endocr. Rev.

[22] Yamauchi T., Kamon J., Ito Y., Tsuchida A., Yokomizo T., Kita S., Sugiyama T., Miyagishi M., Hara K., Tsunoda M. (2003). Cloning of aiponectin receptors that mediate antidiabetic metabolic effects. Nature.

[23] Yang B., Brown K., Chen L., Carrick K., Clifton L., Mc Nulty J., Winegar D., Strum J., Stimpson S., Pahel G. (2004). Serum adiponectin as a biomarker for in vivo PPARgamma activation and PPARgamma agonist-induced efficacy on insulin sensitization/lipid lowering in rats. BMC Pharmacol.

[24] Li C.J., Sun H.W., Zhu F.L., Chen L., Rong Y.Y., Zhang Y., Zhang M. (2007). Local adiponectin treatment reduces atherosclerotic plaque size in rabbits. J. Endocrinol.

[25] Menzaghi C., Trischitta V., Doria A. (2007). Genetic Influences of Adiponectin on Insulin Resistance, Type 2 Diabetes, and Cardiovascular Disease. Diabetes.

[26] Garaulet M., Viguerie N., Porubsky S., Klimcakova E., Clement K., Langin D., Stich V. (2004). Adiponectin Gene Expression and Plasma Values in Obese Women during Very-Low-Calorie Diet. Relationship with Cardiovascular Risk Factors and Insulin Resistance. J. Clin. Endocrinol. Metab.

